# Well-Being Elective in Health Professional Education: A Pilot Study

**DOI:** 10.3390/pharmacy14060128

**Published:** 2026-09-01

**Authors:** Desirée N. Shapiro, Nikki Ashtiani, Raphael Cuomo, Candis M. Morello

**Affiliations:** 1 San Diego School of Medicine, University of California, La Jolla, CA 92093, USA; 2 San Diego Skaggs School of Pharmacy and Pharmaceutical Sciences, University of California, La Jolla, CA 92093, USA

**Keywords:** well-being, pharmacy, well-being course, education, empathy, compassion, health professional students, wellness intervention, mental health

## Abstract

**Background:** Health professional training is inherently challenging, with elevated rates of burnout, stress, and diminished well-being. Dedicating curricular time to well-being education may build awareness and skills to help students navigate these demands. **Methods:** A 10-week well-being elective was designed and implemented for health professional students. Participants in the intervention group were predominantly pharmacy students. This pilot feasibility study used a pre- and post-intervention survey design with a comparison group. Course evaluation, self-reported confidence, knowledge, skills, and attitudes about well-being practices, and measures of empathy, compassion, stress, social support, and burnout were assessed [*n* = 29 intervention, *n* = 24 comparison]. **Results:** Students in the elective reported high satisfaction in the course and significantly greater improvements than those in the comparison group in confidence applying wellness strategies in their personal and academic lives, identifying mental health and wellness resources, preparing nourishing meals, showing up authentically, using mindfulness, practicing self-compassion, as well as in frequency of reflecting on wellness, practicing mindfulness and self-compassion, and applying wellness strategies (all *p* < 0.05). Prioritization of wellness and feelings of social connection also improved significantly more than in the comparison group. At 6-week delayed follow-up, participants reported significantly greater increases in confidence in preparing nourishing meals, showing up authentically, applying mindfulness, as well as in frequency of mindfulness practice, reflection on wellness, and greater gains in social connection (all *p* < 0.05). No significant between-group differences were observed on the validated measures of empathy, burnout, or self-compassion. **Conclusions:** A novel well-being elective designed for health professional students significantly improved select well-being-promoting behaviors and emotional connectedness among participating students. Scalable curricula may bolster student well-being and foster community in demanding academic environments.

## 1. Introduction

Health professional students are vulnerable to concerning rates of psychological distress, suicidal ideation, and burnout, as well as declines in empathy over the course of training [1,2,3,4]. Prioritizing the well-being of healthcare trainees and professionals through evidence-based curricula may mitigate burnout, improve experience during training, and empower students to optimize their empathy and compassion, therefore improving patient care and workplace culture [5].

The existing literature supports integration of well-being, empathy, and compassion into health professional curricula. Research links enhanced well-being, empathy, and compassion with burnout reduction [6]. Wellness and mind–body focused interventions do not eliminate student stressors but may bolster their resilience and ability to cope with stressors [7]. Well-being curricula that build awareness of stress management strategies, reduce burnout, and cultivate empathy and compassion for self, patients, and peers are underutilized in health professional curricula. Proactively teaching and discussing risk factors, including fatigue, burnout, interpersonal conflict, emotional exhaustion, and neglect of physical and mental health, is essential for healthcare trainees.

Students are also seeking spaces to learn and develop their understanding of well-being and have requested that well-being electives and initiatives be implemented during medical training [8]. Mental health stigma among pharmacy students is associated with reduced help-seeking, suggesting this population may particularly benefit from well-being programming [4]. Creating spaces to openly discuss topics of well-being, identity, and empathy has the potential to support trainees through their rigorous programs. Studies show that feeling connected and part of a community during health professional training has direct effects on student well-being and satisfaction [9]. One large-scale implementation of a wellness course for undergraduates was promising for well-being promotion compared to other psychology courses [10].

This study examined the feasibility and impact of a 10-week experiential well-being elective for health professional students. The elective was developed to provide health professional students with the education and tools necessary to manage the demands of health care professional training. By introducing these concepts and tools early in their training, the course aimed to build awareness, build reflective practice, and cultivate skills that students may carry forward throughout their professional development. Our approach centered on integrating evidence-based well-being strategies with relevant background knowledge to equip students with a practical, accessible, and personalized toolkit. We also addressed the challenges of navigating professional environments and our collective responsibility in fostering inclusive workplaces where individuals can express their authentic selves without fear of judgment or discrimination; these are factors that contribute to cognitive fatigue, decreased performance, and burnout [11].

## 2. Materials and Methods

At the University of California San Diego, pharmacy, medical, and public health trainees were recruited via email, newsletter postings, instructor promotions, word-of-mouth, and digital flyers.

Core course topics included the Science of Well-being, Mind–Body Connection, Identity and Authenticity, Self and Community Compassion, Nutrition and Nourishing the Body, Healthy Behavior Change, Play and Joy, and Community and Relationships. Content experts were identified to deliver select sessions. A licensed dietician led a live food demonstration based on student interest and Q&A for the nutrition class. A guest mindfulness expert facilitated interactive meditation and mindfulness exercises for the mindfulness session.

Each session opened with a routine wellness check-in and the intentional creation of a welcoming environment. Following the welcome routine, sessions incorporated a combination of instruction and knowledge sharing, transitioning into interactive small group and individual reflection activities. Students completed weekly self-reflective assignments and a final project on well-being promotion. Students were also invited to optional 20 min 1:1 well-being check-in calls, which allowed students to reflect on their well-being and brainstorm ways to incorporate well-being practices into their unique schedules.

Students were not randomized to condition, as enrollment in the elective was voluntary. Pharmacy students with no prior enrollment in the elective were randomly sampled and invited to serve as a comparison group. Because a substantial proportion of the P1 class enrolled in the elective, the comparison group consisted largely of P2 students. Surveys were collected at baseline, immediate post-intervention (10 weeks), and delayed follow-up (6 weeks), using the Qualtrics email option to distribute surveys. Participants were given time to complete surveys in class to maximize the likelihood of completion, each of which took approximately 10–15 min. Students in the comparison group were sent the surveys via email.

Self-reported measures of well-being practices, attitudes, confidence, knowledge, and skills, along with the Toronto Empathy Questionnaire, Self-Compassion Scale–Short Form, Perceived Stress Scale, WHO-5 Well-Being Index, Inclusion of Other in the Self Scale, and Inclusion of Community in Self Scale. Appendix A provides details on each measure. Demographic information, including age, sex assigned at birth, sexual orientation, year in training, gender identity, race/ethnicity, and spiritual/religious orientation, was collected in the pre-survey to characterize the sample. The post-intervention assessment additionally included items on satisfaction and experience in the elective. Participants generated their own identification code at each timepoint, and responses were linked across timepoints by exact match on that code. Analyses used complete cases, defined as participants whose responses could be linked across the two relevant timepoints. Because participants generated their own study identifiers, a subset of baseline and follow-up responses could not be reliably matched and were excluded rather than entered. Complete-case samples included 29 intervention and 24 comparison participants for baseline-to-immediate-post comparisons, and 27 intervention and 24 comparison participants for baseline-to-six-week follow-up comparisons. The difference in means was then calculated for students who completed both pre- and post-surveys to assess changes in scores over time. For participants with data at both time points, change scores were calculated at the individual level, and the mean change was compared between groups using two-sided Welch independent-samples *t*-tests for baseline-to-immediate post and baseline-to-6-week follow-up. Immediate-post-to-6-week changes were not analyzed.

Analyses were conducted in Python (3.12.0) using pandas and SciPy by calculating participant-level change scores and doing complete-case analysis comparing changes between groups with two-sided Welch independent-samples *t*-tests. No one-to-one matching of participants to comparisons was performed.

Intervention students were also invited via a widely distributed email to the cohort to participate in a post-intervention focus group, including questions and discussion regarding their overall experience in the course, the usefulness and relevance of the intervention, which content areas were most useful, and how the overall experience of the study was perceived. Five students were able to attend the focus group at the end of the quarter to share feedback with facilitators regarding their experience in the course.

This study received exempt status from the University of California, San Diego Office of IRB Administration (Protocol #810033). The exemption was granted on the basis that the research was conducted within an established educational setting involving normal educational practices, did not adversely affect students’ learning or the evaluation of instructors, and posed no reasonable risk to participants through disclosure of their responses. Informed consent for participation was obtained from all of the participants, and the study adhered to all the IRB board’s ethical standards and regulations.

## 3. Results

A total of 42 students participated in the 10-week well-being elective in the winter of 2025, the majority of whom were in their first year of pharmacy school. With approximately 76 students per pharmacy school class, this was a substantial proportion of the first-year class electing to take this course. All 42 enrolled students completed the end-of-course survey. Because participants generated their own study identifiers, baseline responses could only be linked to follow-up responses for 29 of the 42 intervention students; the remaining responses could not be matched with confidence and were excluded from the change score analyses. A comparison group of students was recruited to complete assessments. Of the 35 in the comparison group, 24 had linkable baseline and immediate post responses and 24 had linkable baseline and delayed post responses.

Demographic characteristics were collected in the baseline survey and are available for the 29 intervention students with linked baseline data (Table 1). Of these, 89.7% self-identified as female, 10.3% identified as male, and 3.4% identified as either a trans man, gender queer, gender non-conforming, nonbinary, or other. Additionally, 75.9% of participants identified as Asian, 31.0% identified as White, 10.3% identified as Hispanic or Latinx, and 3.4% identified as Native American (Table 1). Percentages exceed 100% because participants could select multiple categories.

All 42 of the health professional students who participated in the elective completed the end-of-course evaluation. All students (100%) were satisfied with their experience in the course. Ninety-eight percent of the students reported recommending it to other students, with one selecting neutral; 93% reported they were more likely to dedicate time to their wellness because of this elective; over 95% felt the course added to their wellness; and 98% found the course useful to their personal lives. Participants in the intervention group reported significantly greater improvements than those in the comparison group in confidence applying wellness strategies in their personal and academic lives (change of +5.86 vs. +0.88; *p* = 0.0052), identifying mental health and wellness resources (+6.14 vs. −0.17; *p* = 0.0042), preparing nourishing meals (+6.62 vs. −1.21; *p* = 0.0001), showing up authentically (+5.14 vs. −1.71; *p* = 0.0014), using mindfulness (+5.38 vs. +0.21; *p* = 0.0021), and practicing self-compassion (+5.45 vs. +0.25; *p* = 0.0017). They also showed greater increases in frequency of reflecting on wellness (+2.86 vs. −1.88; *p* = 0.0092), practicing mindfulness (+3.28 vs. −1.13; *p* = 0.0092), practicing self-compassion (+4.34 vs. +0.08; *p* = 0.0054), and applying wellness strategies (+3.79 vs. −0.42; *p* = 0.0311). Prioritization of wellness improved more in the intervention group (+1.38 vs. +0.08; *p* = 0.0071), as did feelings of social connection, with a greater reduction in the perception that others “barely know me” (+0.31 vs. −0.21; *p* = 0.0247).

At delayed follow-up, participants in the intervention group demonstrated significantly greater improvements than those in the comparison group in several domains of wellness. These included increased confidence in preparing nourishing meals (change of +3.93 vs. −1.33; *p* = 0.0017), showing up authentically (+2.78 vs. −2.21; *p* = 0.0023), and using mindfulness (+2.37 vs. −0.83; *p* = 0.0468). Frequency of mindfulness practice also improved more among intervention participants (+2.00 vs. −1.00; *p* = 0.0431), as did reflection on wellness (+2.48 vs. −1.71; *p* = 0.0479). Additional benefits were observed in social and self-related domains, including a greater reduction in the perception that “people barely know me” (+0.30 vs. −0.38; *p* = 0.0327) and increased identification with others (+0.44 vs. −0.21; *p* = 0.0364).

**Figure 1:**
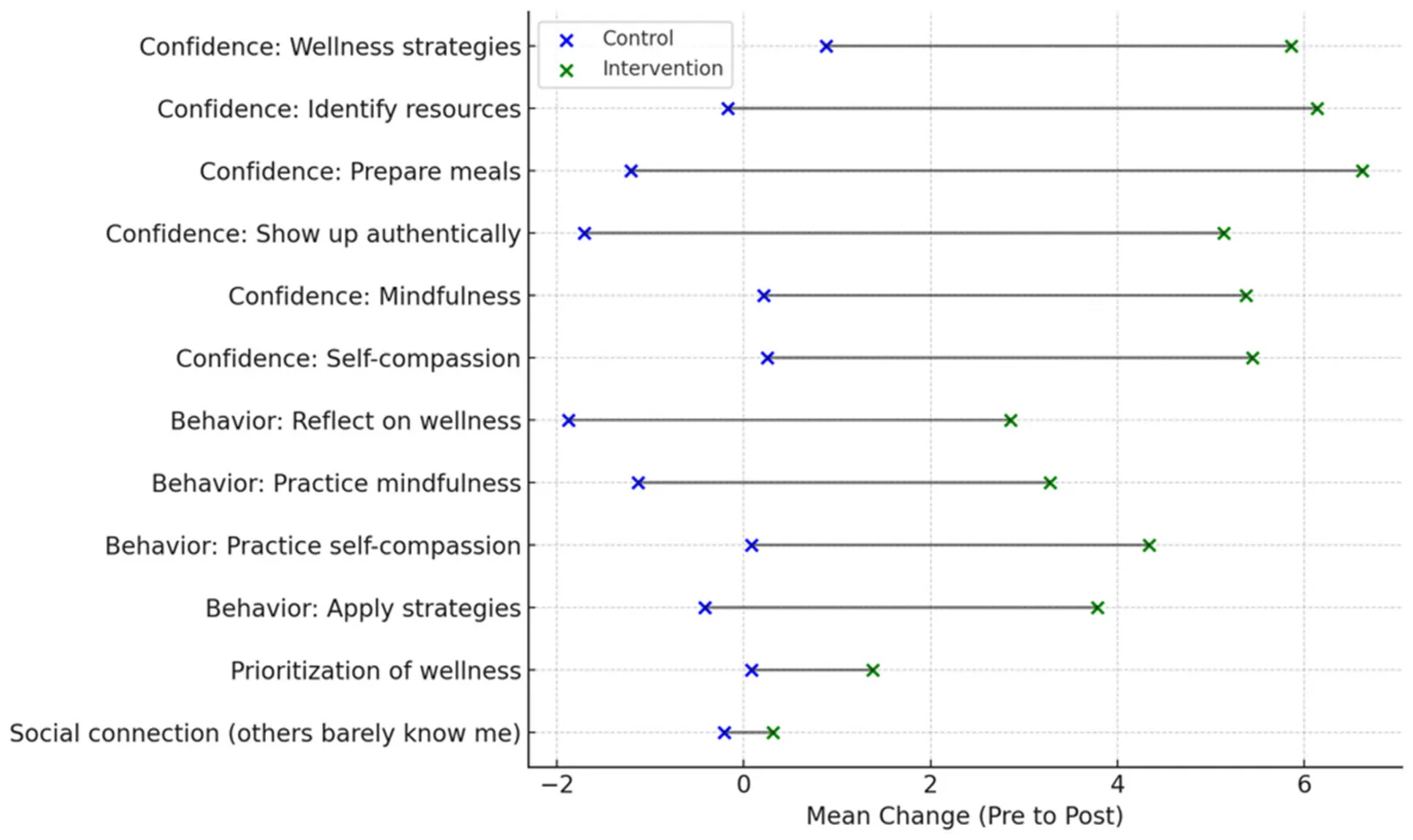
Statistically significant differences in pre-post changes between intervention and comparison groups for wellness-related outcomes for health professional participants. Each point represents the mean change from baseline to immediate post-intervention, by group, across domains including wellness strategy confidence, wellness behavior frequency, and social connectedness. Confidence items are on a 1–10 scale, with all other question responses on a 1–5 scale.

Overall, as displayed in Figure 1, the intervention group showed enhancement across multiple dimensions of wellness and connection. No statistically significant changes were observed on the other scale measures (e.g., empathy, burnout, self-compassion).

Qualitative results from the free-response questionnaire and the focus group indicated that intervention participants thought the course was beneficial in their personal and professional lives, noting that stress reduction, empathy, and compassion connect to their well-being. Participating students further emphasized the significance of peer connection, noting the importance of not feeling alone in their journey and the role of community in supporting their well-being. More details are provided in Appendix B.

## 4. Discussion

This pilot study examined the feasibility and impact of a 10-week well-being elective designed for health professional students at the University of California, San Diego. More than half of the first-year pharmacy class selected to take the elective, demonstrating strong interest in this content and supporting its feasibility. All students met the course requirements, and the majority attended every session, indicating that the course was both acceptable and manageable within the existing curriculum. Furthermore, students completed the assigned coursework and actively engaged in small-group discussions, providing additional evidence of the feasibility and successful implementation of the course. Students in the elective reported utilizing the skills learned and rated their experience with high satisfaction, which is encouraging. This is particularly true given that high-achieving individuals do not always view help-seeking as accessible or relevant to themselves, and may perceive it as threatening to their career given the persistent stigma surrounding mental health in professional training [10].

Compared to students who did not take the elective course, students who participated in the elective demonstrated greater confidence implementing wellness skills, including preparing nourishing meals, showing up authentically, identifying resources, using mindfulness, and practicing self-compassion, as well as improved feelings of social connection. Students who were in the elective reported more frequent practice of mindfulness and self-compassion, reflection on well-being, and applying wellness strategies. Those who took the elective reported prioritizing their wellness more and feeling more socially connected. Confidence and frequency both improved together for the above measures, suggesting real behavior change, not just an attitude shift. Meal-prep confidence showed the largest, most durable effect, indicating a concrete, teachable skill essential for health. Social connection gains grew stronger at the 6-week follow-up rather than fading, an interesting finding that warrants additional exploration given the importance of community and belonging in student success and well-being. Most gains persisted at 6-weeks after the elective ended, so this brief elective shows a signal for continued benefit. The comparison group showed a decline in self-reported measures over time. This may indicate that this elective not only added immediate benefit but also reversed a negative trajectory. Focus group findings revealed themes of reduced stress, increased well-being behaviors, and enhanced compassion, which participants attributed directly to the elective’s content, experiential learning, and sense of community.

This study had several limitations and potential biases that may affect generalizability. Although statistically significant changes were observed in confidence and wellness behaviors (Figure 1), no significant between-group differences emerged on measures of burnout, empathy, compassion, well-being, and stress. This may have been linked to limited power to detect change on these broader constructs, whereas measures specific to the intervention, more closely aligned with the brief curriculum focused on skills, reached significance. Enrollment was voluntary and not randomized. Students who chose to enroll may have had a greater preexisting interest in or engagement with practices related to wellness, which could have influenced both participation and outcomes. Alternatively, some students may have recognized this need in themselves and sought out the elective for that reason. Looking at how much students changed, rather than where they ended up, helps address the concern that one group simply started off more engaged with wellness. It does not, however, rule out the possibility that students who chose to enroll were already more ready or motivated to change, and that this readiness itself, rather than the curriculum, could explain some of the improvement observed. A further limitation was the lack of a formal baseline comparability analysis. The comparison group differed from the intervention group in year of training, as most intervention participants were first-year pharmacy students and most comparison participants were in their second year. Future studies should incorporate designs that allow for a more rigorous assessment of baseline comparability. Identifiers generated by participants could not be matched for all of the intervention participants. In addition, we could not control for the assessment conditions and the external stressors inherent to health professional training, all of which may have further influenced results. Although the elective was open to all health professional students, there was a majority of pharmacy students enrolled in this elective, limiting generalizability to all health professional students. Finally, this study was not able to distinguish the specific effects of the instructor’s approach from those of the curriculum itself.

Health professional students face significant pressures including mental health struggles, burnout, and suicidal ideation, making it increasingly important to equip this population with the information and skills and sustain their well-being throughout training and beyond [12]. As academic competition intensifies, feelings of isolation often increase, leading many students to rely on maladaptive coping strategies and perfectionist tendencies that perpetuate cycles of burnout and mental health decline. A significant mental health burden has been documented among pharmacy students globally, prompting calls for comprehensive initiatives encompassing early identification and screening, access to support services, resilience-building interventions, and stress management programming [12]. As pharmacy students comprised the majority of participants in this study, these findings highlight the particular relevance of early awareness and proactive support for this population.

A central component of the curriculum was the intentional cultivation of psychological safety, belonging, and community, while creating a space for students to learn, reflect, and connect with peers in a noncompetitive and collaborative environment. This deliberate reduction in competition and emphasis on peer connection directly addressed one of the core drivers of student distress, offering a rare opportunity to engage authentically without the pressures that typically characterize health professional training. The incorporation of time management skills, meaning-making, and purpose reflection further addressed dimensions identified in the literature as impactful on student well-being in health professional programs [13].

Future work should address these challenges by strengthening research design, increasing the sample size, and expanding the population. In addition, it is important to consider the optimal dose of the experience and identify sensitive measurement tools capable of capturing individual differences across a broader range of well-being dimensions to evaluate the impact of structured well-being curricula in health professional education.

## 5. Conclusions

This well-being elective course for health professional students was successfully implemented, achieving high levels of student satisfaction and strong endorsement to other students. Those who completed the elective showed improved confidence in identifying resources, using mindfulness, practicing self-compassion, preparing nourishing meals, applying wellness strategies, and engaging authentically in their personal and academic lives. Additionally, they showed increased frequency of reflecting on wellness and practicing mindfulness, self-compassion, and wellness strategies, as well as gains in prioritizing wellness and social connection.

The findings of this study support the value of continued access to focused well-being programming throughout rigorous health professional training and suggest such offerings warrant consideration within health professional curricula, especially among pharmacy education. Further research is needed to identify the most effective and feasible approaches to integrating well-being education within existing curricular demands and to evaluate long-term impact across diverse student populations.

## Figures and Tables

**Table 1:** Demographics of intervention and comparison groups with complete pre/post data.

Characteristic	Category	Intervention (*n* = 29), *n* (%)	Comparison (*n* = 24), *n* (%)
School	Pharmacy (Skaggs)	28 (96.6%)	24 (100.0%)
	Public health	1 (3.4%)	0 (0.0%)
Year in Training	First year	28 (96.6%)	10 (41.7%)
	Second year	0 (0.0%)	14 (58.3%)
	Third year	1 (3.4%)	0 (0.0%)
Age Group	18–24 years	25 (86.2%)	20 (83.3%)
	25–34 years	4 (13.8%)	4 (16.7%)
Sex Assigned at Birth	Female	26 (89.7%)	15 (62.5%)
	Male	3 (10.3%)	9 (37.5%)
Race or ethnicity *	Asian	22 (75.9%)	16 (66.7%)
	White	9 (31.0%)	5 (20.8%)
	Hispanic or Latinx	3 (10.3%)	3 (12.5%)
	Native American	1 (3.4%)	0 (0.0%)
	Pacific Islander	0 (0.0%)	0 (0.0%)
	Self-identify (Middle Eastern)	0 (0.0%)	1 (4.2%)
	Prefer not to answer	0 (0.0%)	1 (4.2%)
Gender Identity *	Female	26 (89.7%)	15 (62.5%)
	Male	3 (10.3%)	9 (37.5%)
	Trans male/trans man	1 (3.4%)	0 (0.0%)
	Gender queer	1 (3.4%)	0 (0.0%)
	Gender non-conforming	1 (3.4%)	0 (0.0%)
	Nonbinary	1 (3.4%)	0 (0.0%)
	Other	1 (3.4%)	0 (0.0%)
	Prefer not to answer	1 (3.4%)	0 (0.0%)

Note. * race or ethnicity and gender identity allowed multiple selections, so totals may exceed group N or 100%.

## Data Availability

The data presented in this study are available on request from the corresponding author due to the privacy of qualitative student responses.

## Abbreviations

The following abbreviations are used in this manuscript:

TEQ	Toronto Empathy Questionnaire
SCS-SF	Self-Compassion Scale Short Form
IOS	Inclusion of Other in the Self Scale
ICS	Inclusion of Community in Self Scale
PSS-10	Perceived Stress Scale
WHO-5	World Health Organization-Five Well-Being Index

## Appendix A

**Table A1:** Validated measures used to evaluate well-being metrics in the well-being elective course pre- and post-surveys.

Metric	Validated Survey Measure
Empathy	Toronto Empathy Questionnaire (TEQ) is a brief and valid instrument for the assessment of empathy [12].
Self-Compassion	Self-Compassion Scale Short Form measures the self-acceptance and self-kindness components of mindfulness. It is a tool proven to be a reliable and sound instrument in assessing self-compassion [13].
Belonging and Connectedness	Inclusion of Other in the Self (IOS) Scale and Inclusion of Community in Self (ICS) Scale [14,15] and Watts Connectedness Scale [16].
Social Isolation	PROMIS Item Bank v2.0—Social Isolation—Short form 6a [17].
Stress	Perceived Stress Scale (PSS-10), a 10-item questionnaire with extensive research proving valid, reliable psychometric properties across diverse demographics [18].
Burnout	Single-item measures of emotional exhaustion and depersonalization that demonstrated concurrent validity with the full MBI [19].
Well-Being	The World Health Organization’s Five Well-Being Index (WHO-5) is a short self-reported measure of current mental well-being. The WHO-5 has been found to have good construct validity as a unidimensional scale measuring well-being in studied populations [20,21,22,23].

## Appendix B

“After taking this elective, I realize that I have the ability to incorporate little habits in my every day life that can boost my well-being exponentially. I never realized how hard I was on myself and this class helped me realize that I had a community of people that may be struggling with the same thing and I can reach out to them. I also learned that prioritizing wellness, building compassion, and practicing empathy doesn’t have to be a big task. They can be little things we remind ourselves of and practice every day or week that can help us in the long run.”
“I gained a deeper understanding of my emotions and thought patterns [and] helped me build self awareness and self compassion. I also learned how to manage stress during busy school life. The course also enhances my ability to empathize with others, foster gratitude, and cultivate a positive mindset that can help me live a more balanced and positive life”
“This elective has taught me how to take better care of myself mentally. I started caring more for my well-being and ensuring that I take care of my physical and mental health. This class has taught me the importance of self-care and to also listen to my body’s wants and needs.”
“I take away the importance of incorporating well-being into my day-to-day life. There are certain strategies that have stuck with me, like box-breathing and eating balanced meals. Also, this class has pushed me to make an active effort to connect with my community and reach out to people.”
“It’s important to take care of your wellbeing in order to be a productive and compassionate community member. There are many simple ways to incorporate wellbeing practices into your life whether through meditation, nutrition, sleep, or physical activity. I am motivated to start prioritizing these practices more in my daily life.”
“I’ve learned that taking care of my own well-being is just as important as caring for others. This elective reminded me that empathy and compassion aren’t just feelings—they are actions that can make a difference in both patient care and daily life. I’ll take away the importance of listening without judgment, being present, and showing kindness, even in small moments. It also taught me that showing compassion to myself helps me stay balanced [to] continue supporting others.”
“I know how to be more compassionate towards myself, as well as others. Seeing my classmates open up made me feel more connected and relatable to them. I will also prioritize making time to do things that make me happy, and spending time with quality people.”
